# Response to electroconvulsive therapy is associated with a more diverse oral microbiome– a prospective longitudinal cohort pilot study

**DOI:** 10.1007/s00406-025-01976-3

**Published:** 2025-02-14

**Authors:** Christoph Ammer-Herrmenau, Jacob Hamm, Albrecht Neesse, Kilian Günther, Matthias Besse, David Zilles-Wegner

**Affiliations:** 1 Department of Gastroenterology, Gastrointestinal Oncology and Endocrinology, University Medical Center Göttingen, Göttingen, Germany; 2 Department of Psychiatry and Psychotherapy, University Medical Center Göttingen, Von-Siebold-Str. 5, 37075 Göttingen, Germany

**Keywords:** Electroconvulsive therapy, Treatment resistance, Depression, Oral microbiome, Oxford nanopore technologies

## Abstract

Recently it has been shown that psychiatric disorders are associated with changes in the host microbiome. Little is known about the association of electroconvulsive therapy (ECT) and microbiome alterations. In our pilot-study, 15 patients with severe or treatment resistant depression were prospectively recruited and oral swabs were collected pre- and post-ECT. Compared to a control group, ECT did not lead to a significant microbial shift in longitudinal samples (*p* = 0.65). However, alpha diversity measurements significantly differed between responders and non-responders before ECT (observed species *p* = 0.014, Shannon *p* = 0.03) and after ECT (observed species *p* = 0.015, Shannon *p* = 0.13).

**Supplementary Information:**

The online version contains supplementary material available at 10.1007/s00406-025-01976-3.

## Introduction

Major depressive disorder is a frequent and often disabling condition, which largely contributes to the burden of disease [1]. Pathophysiology of depression involves a complex interplay of genetic and environmental factors. Several lines of evidence suggest a role for inflammatory processes [2] in a substantial proportion of but not all patients with depression [3]. Moreover, the human microbiome and so-called microbiota-gut-brain axis have been implicated in the (dys-)regulation of inflammatory processes [4]. In the last decade, this area of research has gained much attention in the context of neuropsychiatric disorders [5, 6]. A systematic review found disparities in α-diversity (diversity within an individual or group) and β-diversity (diversity between groups) of the microbiota in subjects with depression compared to healthy controls [7]. A randomized controlled trial with multi-strain probiotic supplement versus placebo in addition to treatment-as-usual led to a stronger decrease of depressive symptoms in the probiotics group [8]. A systematic review of animal and human studies concluded that Fecal Microbiota Transplant (FMT) may lead to a reduction of depressive symptoms [9]. Thus, the microbiome may both play a role in the pathophysiology of depressive disorders and could be a potential target for interventions in addition to standard treatments.

Electroconvulsive therapy (ECT) is an effective treatment for several neuropsychiatric conditions, and severe depressive disorders are the most common indications in western countries [10]. The mechanism of action comprises neuroplasticity/neurotrophic effects [11–13] and modifications of immunological/inflammatory processes [14]. Beyond established clinical predictors of treatment response like higher age and presence of psychotic or psychomotor symptoms [15], recent studies aim to detect biological markers that may ultimately lead to clinically applicable tools that are able to guide treatment decisions [16]. E.g., ECT responders showed a significantly lower expression of microRNA miR-223-3p in comparison to non-responders. Together with higher expression levels of several proinflammatory factors (IL-6, IL-1b, TNF-α, NLRP3) this suggests the existence of distinct subgroups of depressive disorders with a particular good response to ECT [17]. In contrast to the existing literature on inflammatory cytokines, we are not aware of any studies investigating microbiome composition in patients treated with ECT for depression [18, 19].

The aim of our study was therefore to investigate the microbiome in patients with severe and treatment-resistant depressive disorders during the course of an ECT series. Using an exploratory approach, our study aims to provide first evidence on the following questions:

- is there a change in the microbiome in patients with unipolar depression treated with ECT?
- do ECT responders and non-responders differ in their microbiome composition before or after the treatment?
- can the microbiome before ECT help to predict treatment response to ECT?

## Methods

### Patient cohort

Patients with a diagnosis of treatment resistant depression (F32 or F33 according to ICD-10) were prospectively enrolled at the University Medical Center Goettingen from February 2022 to January 2024. The study was approved by the local ethics committee of the University Medical Center Goettingen (protocol number 14/8/21) and was performed in accordance with the ethical standards laid down in the 1964 Declaration of Helsinki and its later amendments. After obtaining written informed consent, buccal swabs were collected in a highly standardized manner as described previously by our group [20, 21]. In brief, all swabs were frozen at -80 °C within 30 min after collection. Prior to sample collection, patients did not eat, smoke or brush their teeth for at least two hours. Fifteen patients underwent ECT (three times a week for a total of 6–18 sessions up to the discretion of the responsible psychiatrist), and buccal swabs were collected before the first and after the last ECT session. ECT was performed under anaesthesia with Methohexital or Esketamine/Propofol and relaxation with Succinylcholine. As control group, we recruited nine patients with depression (F32 and F33) without any changes in pharmacological treatment between the two buccal swabs collected four weeks apart.

### Microbiome analysis

A comprehensive wet-lab and bioinformatical workflow for microbiome analysis with Oxford Nanopore Technologies (ONT) was previously published by our group [20]. DNA from swabs was extracted using the PureLink microbiome kit (Invitrogen) and subsequently underwent 16 S rRNA PCR, using SQK-16S114-24 (ONT) amplifying the whole 16 S rRNA gene. Utilizing R10.4.1 flow cells all samples were sequenced with a GridION (ONT) and controlled with MinKNOW v.24.02.10. Basecalling was performed with Dorado v.0.7.3 (qscore > 9) and a read-based classification was performed with MetaPont [20]. A library containing all complete NCBI bacterial, archaea, fungal, viral, human and murine RefSeq genomes was built on 10th of September 2020. Subsequent analyses were conducted in R v.4.2.3. Non-bacterial reads were removed and an agglomeration on species level was performed. Decontamination was conducted with decontam using prevalence method with incorporation of negative controls (DNA extraction buffer *n* = 4, PCR controls *n* = 4). Finally, all samples were rarefied to 250.000 reads per sample.

### Statistics

Discrete variables were compared using Fisher exact or Chi-squared test depending on sample size. For continuous variables normality was tested applying Shapiro-Wilk test and consecutively for dichotomous variables Mann-Whitney-U or t-test and for calculations with more than 2 groups Kruskal-Wallis or ANOVA with Dunn-test or Tukey as post-hoc tests were calculated. Two-sided p-values < 0.05 were considered as significant. Differences for beta-diversity (Bray-curtis distance ordinated with principal coordinate analysis (PCoA)) were assessed by PERMANOVA test.

## Results

### Recruitment

**Fig. 1:**
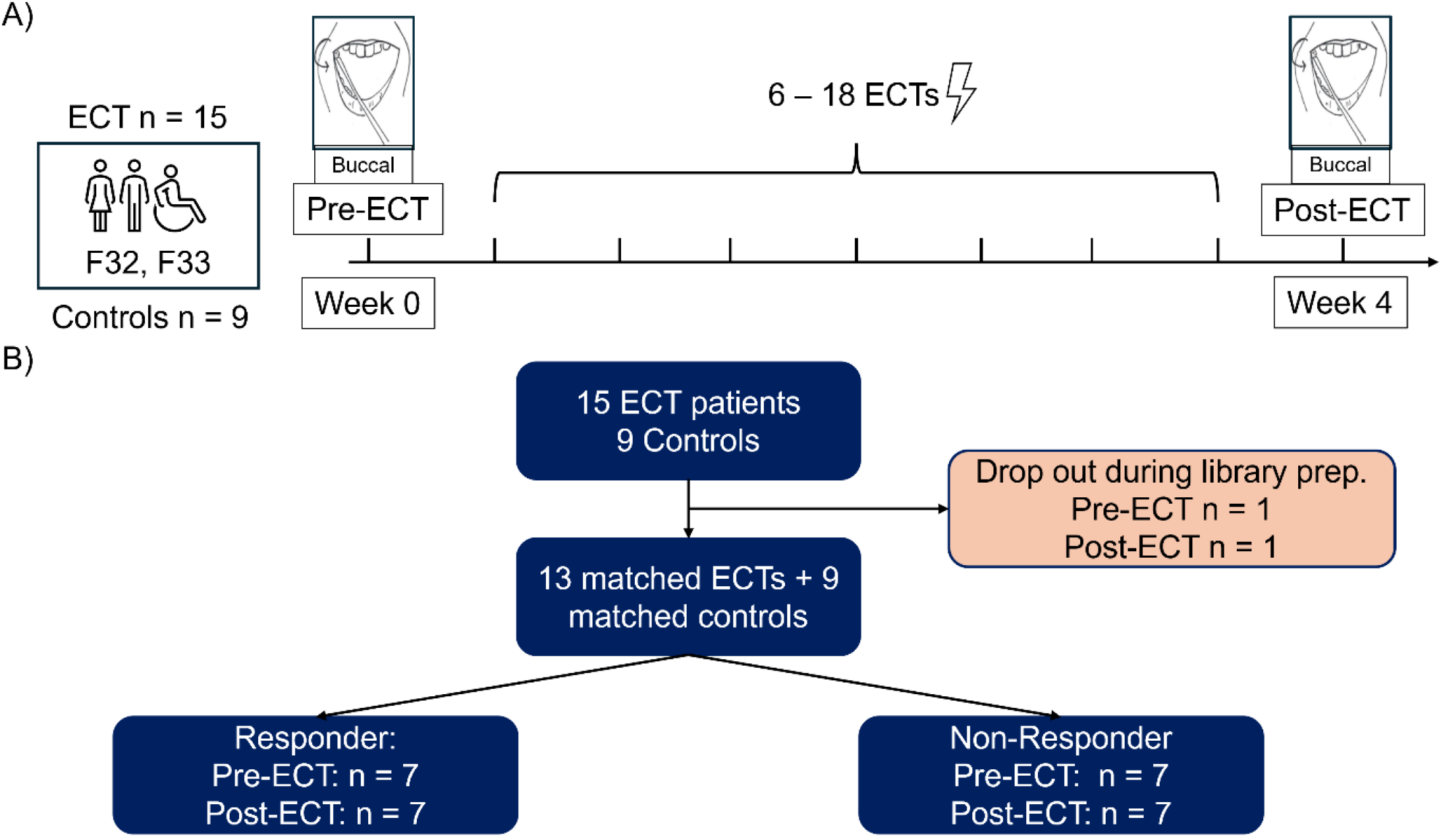
Study design and STROBE flowchart (**A**) Buccal swabs were collected from patients with F32 or F33 diagnosis scheduled for electroconvulsive therapy (ECT) *n* = 15 and without ECT *n* = 9. (**B**) STROBE-flowchart: One pre-ECT and one post-ECT sample from two different patients had to be removed due to failures during the library preparation. Matched samples (pre- and post-ECT) are available for 13 ECT patients and 9 controls. Samples from 7 responders and 7 non-responders were included for pre-ECT and post-ECT cohorts, respectively

Fifteen patients with severe and treatment resistant depression (F33.2 or F33.3 according to ICD-10) receiving a course of ECT were recruited and buccal samples pre-ECT and post-ECT were obtained (Fig. 1A). Buccal samples from patients with depression were collected at intervals of 4 weeks corresponding to mean interval pre- and post-ECT. One pre-ECT and one post-ECT sample was lost during wet-lab preparation. Sufficient microbial data were available from 14 ECT patients (responders *n* = 7) and nine controls, respectively (Fig. 1B).

### Oral microbiome does not alter upon electroconvulsive therapy

To assess whether ECT leads to an oral microbial shift, buccal swabs from patients were collected pre- and post ECT (*n* = 13 matched samples) and from controls. Patients treated with ECT differed significantly from controls regarding symptom severity at baseline as measured by Montgomery–Åsberg Depression Rating Scale (MADRS) and Beck Depression Inventory–II (BDI-II) but not regarding other common microbiome confounders as age, body mass index (BMI), smoking, alcohol consumption or previous medication (Supplementary Table 1). Bray Curtis distances of both time points per individual were compared between controls and ECT revealing no significant differences (Supplementary Fig. 1A). When the ECT cohort was separated into responders and non-responders, the differences between both time-points remained similar to those of the control cohort (Supplementary Fig. 1B).

A more diverse oral microbiome is associated with response to ECT.

**Fig. 2:**
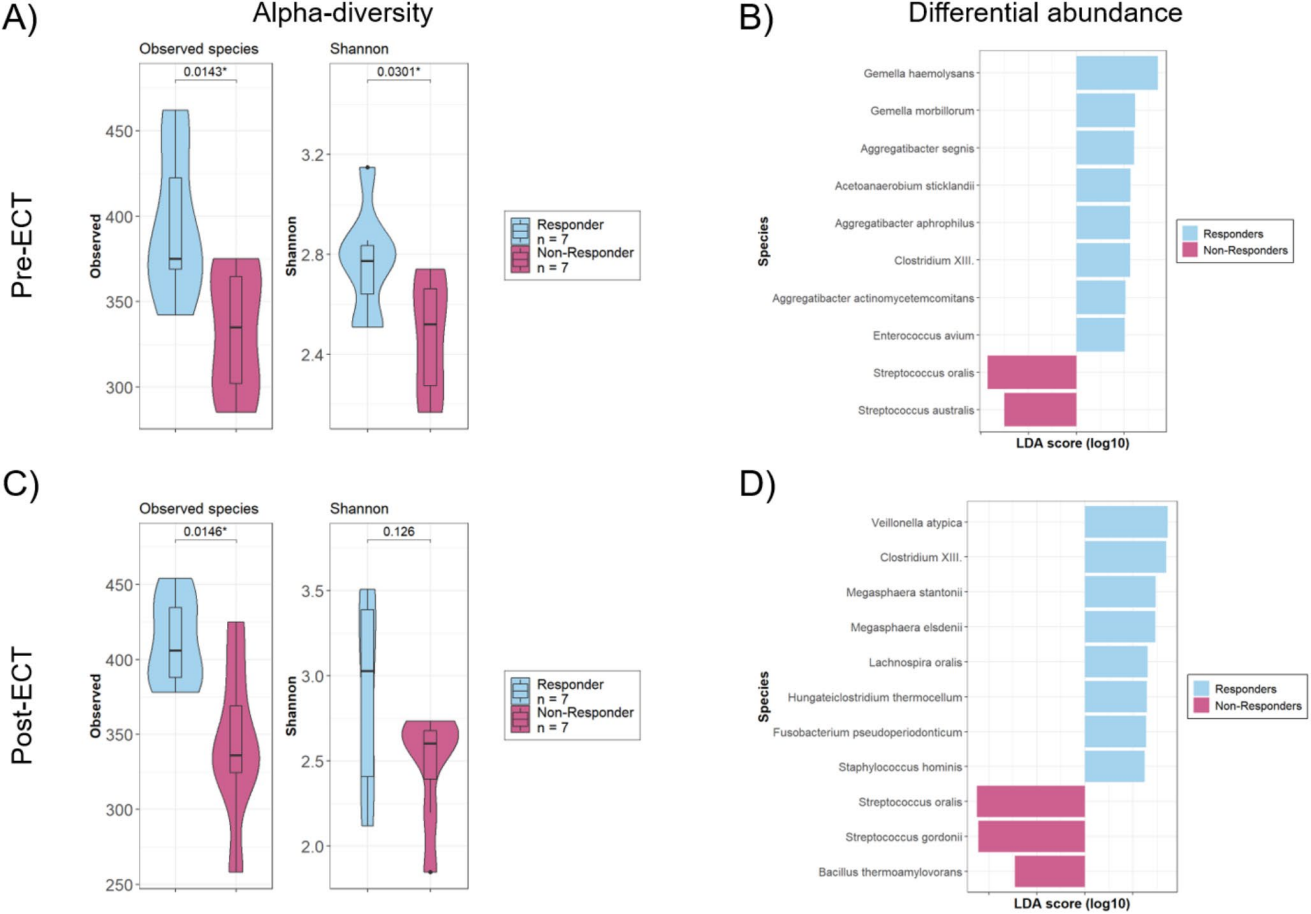
Alpha diversity and differential abundances between electroconvulsive therapy (ECT) responders and non-responders (**A**) Observed species and Shannon index of pre-ECT buccal swabs were compared using t-test. (**B**) Linear discriminant effect size (LEfSe) was applied to determine differential abundant species of responders and non-responders in pre-ECT samples showing species with a linear-discriminant analysis (LDA)-score > 2.5. (**C**) Differences in observed species and Shannon index of post-ECT samples were calculated with t-test and (**D**) differential abundances were assessed with LEfSe. Responders (light blue) and non-responders (pink)

Fourteen patients with microbial samples with sufficient sequencing depth were categorized regarding response to ECT (i.e. decrease in symptom severity ≥ 50%; *n* = 7 per group). Neither group showed any significant differences regarding the abovementioned microbial confounders, though there was a tendency of higher age and more frequent intake of antipsychotics in the responder group (Table 1). Remarkably, the groups did not differ in pre-ECT MADRS and BD-II and ECT related parameters as number of treatments, electrode placement, stimulus dose, and seizure duration. In pre-ECT oral samples both alpha-diversity measurements, observed species (richness), and Shannon index (evenness) were significantly higher in responders compared to non-responders (*p* = 0.014*, *p* = 0.03*). Linear discriminant effect size (LEfSE) revealed ten species as differentially abundant (Fig. 2A-B). Among these, two *Gemella* species, *Gemella haemolysans* and *Gemella morbillorum* and three *Aggregatibacter* species, *Aggregatibacter segnis*, *Aggregatibacter aphrophilus*, *Aggregatibacter actinomycetemcomitans*, were more abundant in the responder cohort and two *Streptococcus* species were dominant in non-responders, *Streptococcus oralis* and *Streptococcus australis*. These results were confirmed in the comparison of post-ECT samples. Here, the observed species but not the Shannon index were significantly different between responders and non-responders (*p* = 0.015*, *p* = 0.13, Fig. 2C). Differential abundance calculation again showed a dominance of the above-mentioned *Streptococcus* species in the non-responder group and *Bacillus thermoamylovorans*, but other differentially abundant species in responders compared to pre-ECT (Fig. 2D). Bray Curtis distances were not significantly different between both groups pre- and post-ECT, respectively (Supplementary Fig. 2).

**Table 1:** Clinical characteristics of responders and non-responders to electroconvulsive therapy (ECT)

Variable	Non-responder, *N* = 7^*1*^	Responder, *N* = 7^*1*^	*p*-value^2^
**Age**	39 (16)	54 (10)	**0.084**
**BMI**	27.4 (5.9)	24.5 (2.3)	0.5
**Sex**			> 0.9
Female	2 / 7 (29%)	2 / 7 (29%)	
Male	5 / 7 (71%)	5 / 7 (71%)	
**Gastrointestinal Diseases**			> 0.9
Chronic constipation	0 / 7 (0%)	1 / 7 (14%)	
No	7 / 7 (100%)	6 / 7 (86%)	
**Liver Diseases**			
No	7 / 7 (100%)	7 / 7 (100%)	
**Cancer Diseases**			
No	7 / 7 (100%)	7 / 7 (100%)	
**Neurological/Psychiatric Diseases**			
No	7 / 7 (100%)	7 / 7 (100%)	
**Diabetes mellitus**	1 / 7 (14%)	0 / 7 (0%)	> 0.9
**Cardiovascular Diseases**	1 / 7 (14%)	1 / 7 (14%)	> 0.9
**HIV**			
No	7 / 7 (100%)	7 / 7 (100%)	
**Rheumatic Diseases**			
No	7 / 7 (100%)	7 / 7 (100%)	
**1st Antidepressant**			> 0.9
SSRI	3 / 7 (43%)	3 / 7 (43%)	
SSNRI	4 / 7 (57%)	3 / 7 (43%)	
Other	0 / 7 (0%)	1 / 7 (14%)	
**2nd Antidepressant**			
No	7 / 7 (100%)	7 / 7 (100%)	
**1st Antipsychotic**			**0.070**
Low-potency antipsychotic	1 / 7 (14%)	0 / 7 (0%)	
Atypical antipsychotic	3 / 7 (43%)	7 / 7 (100%)	
No	3 / 7 (43%)	0 / 7 (0%)	
**2nd Antipsychotic**			0.5
Low-potency antipsychotic	0 / 7 (0%)	1 / 7 (14%)	
Atypical Antipsychotic	2 / 7 (29%)	0 / 7 (0%)	
No	5 / 7 (71%)	6 / 7 (86%)	
**Lithium**	2 / 7 (29%)	1 / 7 (14%)	> 0.9
**Mood stabilisators**			
No	7 / 7 (100%)	7 / 7 (100%)	
**Antidiabetics**			> 0.9
Insulin	1 / 7 (14%)	0 / 7 (0%)	
No	6 / 7 (86%)	7 / 7 (100%)	
**Painkiller**			
No	7 / 7 (100%)	7 / 7 (100%)	
**Antibiotic Intake**			
No	7 / 7 (100%)	7 / 7 (100%)	
**PPI**	2 / 7 (29%)	2 / 7 (29%)	> 0.9
**Immunosuppressive**			
No	7 / 7 (100%)	7 / 7 (100%)	
**Laxative**	0 / 7 (0%)	1 / 7 (14%)	> 0.9
**Statins**			
No	7 / 7 (100%)	7 / 7 (100%)	
**Previous Surgery**			> 0.9
None	6 / 7 (86%)	6 / 7 (86%)	
Bowel surgery	1 / 7 (14%)	0 / 7 (0%)	
Other abdominal surgery	0 / 7 (0%)	1 / 7 (14%)	
**Diagnosis**			0.2
F33.2	7 / 7 (100%)	4 / 7 (57%)	
F33.3	0 / 7 (0%)	3 / 7 (43%)	
**Ethnicity**			> 0.9
Caucasian	6 / 7 (86%)	7 / 7 (100%)	
Other	1 / 7 (14%)	0 / 7 (0%)	
**Diet**			> 0.9
Omnivorous	6 / 7 (86%)	7 / 7 (100%)	
Vegetarian	1 / 7 (14%)	0 / 7 (0%)	
**Daily alcohol consumption**			> 0.9
Never	6 / 7 (86%)	6 / 7 (86%)	
Formerly	1 / 7 (14%)	0 / 7 (0%)	
Currently	0 / 7 (0%)	1 / 7 (14%)	
**Smoking**			> 0.9
No	2 / 7 (29%)	3 / 7 (43%)	
Yes	5 / 7 (71%)	4 / 7 (57%)	
**MADRS pre-ECT**	25.6 (4.9)	30.1 (7.6)	0.3
**MADRS post-ECT**	19.4 (4.3)	10.4 (3.7)	**0.003**
**Difference MADRS**	6 (3)	20 (5)	**0.002**
**BDI-II pre-ECT**	37 (10)	41 (7)	0.3
**BDI-II post-ECT**	28 (16)	9 (7)	**0.025**
**Difference BDI-II**	10 (9)	32 (13)	**0.011**
**Seizure Duration [motor]**	34 (13)	35 (11)	0.8
**Seizure Duration [EEG]**	52 (26)	46 (14)	0.7
**Electrode placement**			0.6
LART (left anterior, right temporal)	3 / 7 (43%)	1 / 7 (14%)	
Change during treatment course	4 / 7 (57%)	6 / 7 (86%)	
**Average stimulation dosage (%**,** 100% = 504 mColoumb)**	50 (32)	57 (13)	0.4
**Anesthetics**			0.5
Methohexital	5 / 7 (71%)	7 / 7 (100%)	
Change during treatment course	2 / 7 (29%)	0 / 7 (0%)	
**Average PSI**	78 (14)	82 (10)	0.7
**Average ASEI**	11,809 (6,408)	10,108 (4,828)	0.5
**Number of ECT sessions**			0.3
≤ 10	1 / 7 (14%)	4 / 7 (57%)	
≥ 11	6 / 7 (86%)	3 / 7 (43%)	
^*1*^Mean (SD); n / N (%)			
^*2*^Mann-Whitney-U test; Fisher’s exact test;			

ASEI– Average seizure energy index, BDI-II– Beck Depression Inventory, MADRS - Montgomery–Åsberg Depression Rating Scale, PSI– Postictal suppression index, SSRI– Selective serotonin reuptake inhibitors, SSNRI– Selective Serotonin-noradrenaline reuptake inhibitors, NSAID– Non-steroidal anti-inflammatory drugs

## Discussion

To the best of our knowledge, our pilot-study shows for the first time an association of the oral microbiome and response to ECT. Though there is a growing body of evidence that psychiatric disorders and their pharmacological and interventional treatment options are linked to the gut microbiome via the brain-gut axis [7], the association with the oral microbiome is heavily understudied. However, the oral microbiome is considered as easily accessible and allows a highly standardized sample collection.

Indeed, our results demonstrate a more diverse pre-ECT oral microbiome may be associated with response to ECT. Remarkably, responders and non-responders did not significantly differ regarding any microbial confounding factor. However, higher age and higher rate of atypical antipsychotics were associated with response to ECT. This phenomenon is in line with existing literature [22].

Notably, *Streptoccocus* species were more abundant in non-responders and *Gemella* and *Aggregatibacter* species were more dominant in responders. These species might be candidates for an oral microbiome classifier to predict response to ECT. Larger cohort studies are warranted to replicate these initial findings. Confirmatory studies should also incorporate biospecimen for microbial metabolites (e.g. saliva or plasma) to further investigate mechanisms how the oral microbiome contributes to ECT responsiveness.

Though the results of this pilot study are promising, there are several limitations of our study. The sample size of this monocentric cohort is small and not sufficient for robust beta-diversity analysis or for the establishment of a microbial classifier. Thus, our results have to be confirmed in larger cohort studies.

However, despite of the small sample size the significant results in α-diversity metrics and differential abundances are highly promising and provide first evidence that the easily accessible oral flora could be exploited as a biomarker for responsiveness to ECT.

## Electronic supplementary material

Below is the link to the electronic supplementary material.

Supplementary Material 1

## Abbreviations

BDI: II–Beck Depression Inventory
BMI: Body mass index
ECT: Electroconvulsive therapy
MADRS: Montgomery–Åsberg Depression Rating Scale
ONT: Oxford Nanopore Technologies

## References

[1] Porst M, Lippe E, von der, Leddin J, Anton A, Wengler A, Breitkreuz J, Schüssel K, Brückner G, Schröder H, Gruhl H, Plaß D, Barnes B, Busch MA, Haller S, Hapke U, Neuhauser H, Reitzle L, Scheidt-Nave C, Schlotmann A, Steppuhn H, Thom J, Ziese T, Rommel A (2022) The Burden of Disease in Germany at the National and Regional Level. Deutsches Arzteblatt Int 119(46):785–792. 10.3238/arztebl.m2022.0314 10.3238/arztebl.m2022.0314 PMC9902892 36350160

[2] Marx W, Manger SH, Blencowe M, Murray G, Ho FY-Y, Lawn S, Blumenthal JA, Schuch F, Stubbs B, Ruusunen A, Desyibelew HD, Dinan TG, Jacka F, Ravindran A, Berk M, O’Neil A (2023) Clinical guidelines for the use of lifestyle-based mental health care in major depressive disorder: World Federation of Societies for Biological Psychiatry (WFSBP) and Australasian Society of Lifestyle Medicine (ASLM) taskforce. World J Biol Psychiatry: Official J World Federation Soc Biol Psychiatry 24(5):333–386. 10.1080/15622975.2022.2112074 10.1080/15622975.2022.2112074 PMC10972571 36202135

[3] Felger JC, Haroon E, Patel TA, Goldsmith DR, Wommack EC, Woolwine BJ, Le N-A, Feinberg R, Tansey MG, Miller AH (2020) What does plasma CRP tell us about peripheral and central inflammation in depression? Mol Psychiatry 25(6):1301–1311. 10.1038/s41380-018-0096-3 29895893 10.1038/s41380-018-0096-3 PMC6291384

[4] Beurel E, Toups M, Nemeroff CB (2020) The bidirectional relationship of depression and inflammation: double trouble. Neuron 107(2):234–256. 10.1016/j.neuron.2020.06.002 32553197 10.1016/j.neuron.2020.06.002 PMC7381373

[5] Cryan JF, O’Riordan KJ, Cowan CSM, Sandhu KV, Bastiaanssen TFS, Boehme M, Codagnone MG, Cussotto S, Fulling C, Golubeva AV, Guzzetta KE, Jaggar M, Long-Smith CM, Lyte JM, Martin JA, Molinero-Perez A, Moloney G, Morelli E, Morillas E, O’Connor R, Cruz-Pereira JS, Peterson VL, Rea K, Ritz NL, Sherwin E, Spichak S, Teichman EM, van de Wouw M, Ventura-Silva AP, Wallace-Fitzsimons SE, Hyland N, Clarke G, Dinan TG (2019) The Microbiota-Gut-Brain Axis. Physiol Rev 99(4):1877–2013. 10.1152/physrev.00018.2018 31460832 10.1152/physrev.00018.2018

[6] Refisch A, Walter M (2023) Die Bedeutung Des Humanen Mikrobioms für die psychische Gesundheit (the importance of the human microbiome for mental health). Nervenarzt 94(11):1001–1009. 10.1007/s00115-023-01552-x 37847418 10.1007/s00115-023-01552-x PMC10620288

[7] Barandouzi ZA, Starkweather AR, Henderson WA, Gyamfi A, Cong XS (2020) Altered composition of Gut Microbiota in Depression: a systematic review. Front Psychiatry 11:541. 10.3389/fpsyt.2020.00541 32587537 10.3389/fpsyt.2020.00541 PMC7299157

[8] Schaub A-C, Schneider E, Vazquez-Castellanos JF, Schweinfurth N, Kettelhack C, Doll JPK, Yamanbaeva G, Mählmann L, Brand S, Beglinger C, Borgwardt S, Raes J, Schmidt A, Lang UE (2022) Clinical, gut microbial and neural effects of a probiotic add-on therapy in depressed patients: a randomized controlled trial. Translational Psychiatry 12(1):227. 10.1038/s41398-022-01977-z 35654766 10.1038/s41398-022-01977-z PMC9163095

[9] Chinna Meyyappan A, Forth E, Wallace CJK, Milev R (2020) Effect of fecal microbiota transplant on symptoms of psychiatric disorders: a systematic review. BMC Psychiatry 20(1):299. 10.1186/s12888-020-02654-5 32539741 10.1186/s12888-020-02654-5 PMC7294648

[10] Espinoza RT, Kellner CH (2022) Electroconvulsive therapy. N Engl J Med 386(7):667–672. 10.1056/NEJMra2034954 35172057 10.1056/NEJMra2034954

[11] Ousdal OT, Brancati GE, Kessler U, Erchinger V, Dale AM, Abbott C, Oltedal L (2022) The Neurobiological effects of Electroconvulsive Therapy studied through magnetic resonance: what have we learned, and where do we go? Biol Psychiatry 91(6):540–549. 10.1016/j.biopsych.2021.05.023 34274106 10.1016/j.biopsych.2021.05.023 PMC8630079

[12] Laroy M, Bouckaert F, Ousdal OT, Dols A, Rhebergen D, van Exel E, van Wingen G, van Waarde J, Verdijk J, Kessler U, Bartsch H, Jorgensen MB, Paulson OB, Nordanskog P, Prudic J, Sienaert P, Vandenbulcke M, Oltedal L, Emsell L (2024) Characterization of gray matter volume changes from one week to 6 months after termination of electroconvulsive therapy in depressed patients. Brain Stimul 17(4):876–886. 10.1016/j.brs.2024.07.015 39059711 10.1016/j.brs.2024.07.015

[13] Sartorius A (2022) Electric field distribution models in ECT research. Mol Psychiatry 27(9):3571–3572. 10.1038/s41380-022-01516-8 35304563 10.1038/s41380-022-01516-8 PMC9708590

[14] Belge J-B, van Eijndhoven P, Mulders PCR (2024) Mechanism of action of ECT in Depression. Curr Top Behav Neurosci 66:279–295. 10.1007/7854_2023_450 37962811 10.1007/7854_2023_450

[15] van Diermen L, Poljac E, van der Mast R, Plasmans K, van den Ameele S, Heijnen W, Birkenhäger T, Schrijvers D, Kamperman A (2020) Toward targeted ECT: the interdependence of predictors of treatment response in Depression further explained. J Clin Psychiatry 82(1). 10.4088/JCP.20m13287 10.4088/JCP.20m13287 33326710

[16] von Mücke-Heim I-A, Pape JC, Grandi NC, Erhardt A, Deussing JM, Binder EB (2024) Multiomics and blood-based biomarkers of electroconvulsive therapy in severe and treatment-resistant depression: study protocol of the DetECT study. Eur Arch Psychiatry Clin NeuroSci 274(3):673–684. 10.1007/s00406-023-01647-1 37644215 10.1007/s00406-023-01647-1 PMC10995021

[17] Kaurani L, Besse M, Methfessel I, Methi A, Zhou J, Pradhan R, Burkhardt S, Kranaster L, Sartorius A, Habel U, Grözinger M, Fischer A, Wiltfang J, Zilles-Wegner D (2023) Baseline levels of mir-223-3p correlate with the effectiveness of electroconvulsive therapy in patients with major depression. Translational Psychiatry 13(1):294. 10.1038/s41398-023-02582-4 37699900 10.1038/s41398-023-02582-4 PMC10497550

[18] Korenblik V, Brouwer ME, Korosi A, Denys D, Bockting CLH, Brul S, Lok A (2023) Are neuromodulation interventions associated with changes in the gut microbiota? A systematic review. Neuropharmacology 223:109318. 10.1016/j.neuropharm.2022.109318 36334762 10.1016/j.neuropharm.2022.109318

[19] Young JR, Evans MK, Hwang J, Kritzer MD, Kellner CH, Weiner RD (2024) Electroconvulsive therapy changes immunological markers in patients with major depressive disorder: a scoping review. J ECT. 10.1097/YCT.0000000000001021 38924480 10.1097/YCT.0000000000001021 PMC11588568

[20] Ammer-Herrmenau C, Pfisterer N, van den Berg T, Gavrilova I, Amanzada A, Singh SK, Khalil A, Alili R, Belda E, Clement K, El Abd A, Gady EE, Haubrock M, Beißbarth T, Ellenrieder V, Neesse A (2021) Comprehensive Wet-Bench and Bioinformatics Workflow for Complex Microbiota Using Oxford Nanopore Technologies. mSystems 6(4):e0075021. 10.1128/mSystems.00750-21 34427527 10.1128/mSystems.00750-21 PMC8407471

[21] Ammer-Herrmenau C, Antweiler KL, Asendorf T, Beyer G, Buchholz SM, Cameron S, Capurso G, Damm M, Dang L, Frost F, Gomes A, Hamm J, Henker R, Hoffmeister A, Meinhardt C, Nawacki L, Nunes V, Panyko A, Pardo C, Phillip V, Pukitis A, Rasch S, Riekstina D, Rinja E, Ruiz-Rebollo ML, Sirtl S, Weingarten M, Sandru V, Woitalla J, Ellenrieder V, Neesse A (2023) Gut microbiota predicts severity and reveals novel metabolic signatures in acute pancreatitis. Gut. 10.1136/gutjnl-2023-330987 10.1136/gutjnl-2023-330987 PMC10894816 38129103

[22] van Diermen L, van den Ameele S, Kamperman AM, Sabbe BCG, Vermeulen T, Schrijvers D, Birkenhäger TK (2018) Prediction of electroconvulsive therapy response and remission in major depression: meta-analysis. Br J Psychiatry: J Mental Sci 212(2):71–80 10.1192/bjp.2017.28 29436330

