# Supplementary material for: Response to electroconvulsive therapy is associated with a more diverse oral microbiome– a prospective longitudinal cohort pilot study
Source: Eur Arch Psychiatry Clin Neurosci. 2025 Feb 14;275(6):1851–8. doi: 10.1007/s00406-025-01976-3 (PMC12500815; doi:10.1007/s00406-025-01976-3)
Supplement: Supplementary file 1 — Supplementary Material 1 [file 406_2025_1976_MOESM1_ESM.docx]

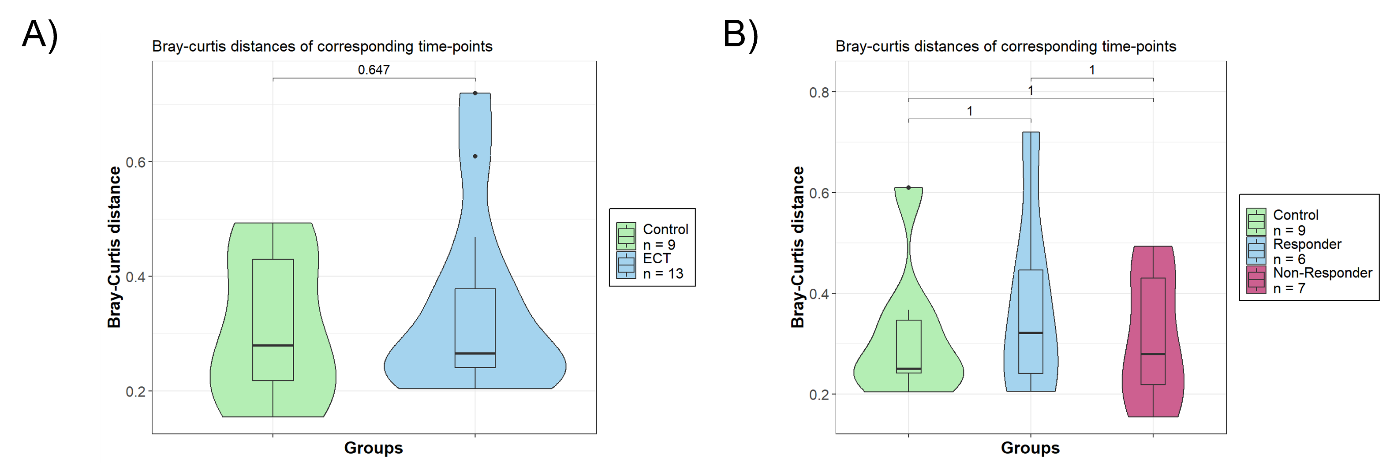


**Supplementary Fig. 1** Bray-curtis distances of corresponding time-points per individual

Bray Curtis distances were calculated per patient for both time-points: Pre-ECT and post-ECT for ECT cohort and week 0 and week 4 for control group. A) Controls were compared to all ECT patients by applying Mann-Whitney-U test. B) Differences between controls, ECT responders and non-responders were obtained by performing Dunn-test and p-values were adjusted with Benjamini-Hochberg correction. ECT – Electroconvulsive therapy


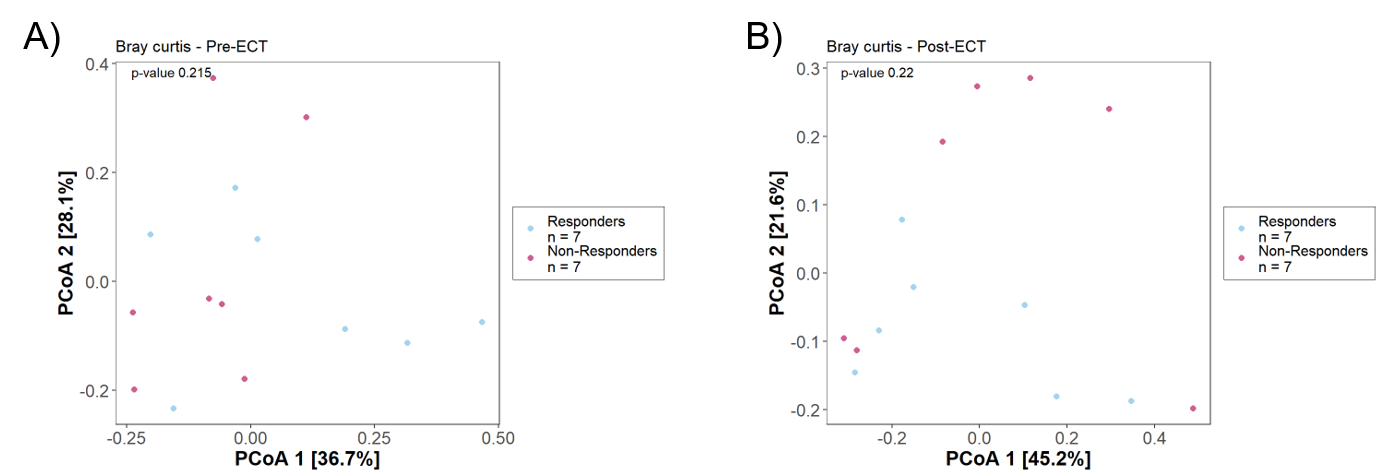


**Supplementary Fig. 2** Bray Curtis distances between responders and non-responders of electroconvulsive therapy (ECT)

Bray Curtis distances were compared of A) oral microbiome samples collected pre-ECT and B) post-ECT between responders and non-responders. Distances are ordinated with principal coordinate analysis (PCoA) and p-values were assessed with PERMANOVA-test.

| **Variable** | **Controls**, N = 9*^1^* | **ECT**, N = 13*^1^* | **p-value***^2^* |
| --- | --- | --- | --- |
| **Age** | 49 (19) | 46 (16) | 0.6 |
| **BMI** | 29.5 (5.5) | 26.1 (4.7) | 0.12 |
| **Sex** |  |  | 0.4 |
| Female | 5 / 9 (56%) | 4 / 13 (31%) |  |
| Male | 4 / 9 (44%) | 9 / 13 (69%) |  |
| **Gastrointestinal Diseases** |  |  | >0.9 |
| Chronic constipation | 0 / 9 (0%) | 1 / 13 (7.7%) |  |
| No | 9 / 9 (100%) | 12 / 13 (92%) |  |
| **Liver Diseases** |  |  |  |
| No | 9 / 9 (100%) | 13 / 13 (100%) |  |
| **Cancer Diseases** |  |  |  |
| No | 9 / 9 (100%) | 13 / 13 (100%) |  |
| **Neurological/Psychiatric Diseases** |  |  |  |
| No | 9 / 9 (100%) | 13 / 13 (100%) |  |
| **Diabetes mellitus** | 1 / 9 (11%) | 1 / 13 (7.7%) | >0.9 |
| **Cardiovascular Diseases** | 3 / 9 (33%) | 2 / 13 (15%) | 0.6 |
| **HIV** |  |  |  |
| No | 9 / 9 (100%) | 13 / 13 (100%) |  |
| **Rheumatic Diseases** |  |  |  |
| No | 9 / 9 (100%) | 13 / 13 (100%) |  |
| **1^st^ Antidepressant** |  |  | 0.8 |
| SSRI | 2 / 9 (22%) | 5 / 13 (38%) |  |
| SSNRI | 6 / 9 (67%) | 7 / 13 (54%) |  |
| Other | 1 / 9 (11%) | 1 / 13 (7.7%) |  |
| **2^nd^ Antidepressant** |  |  | 0.2 |
| Other | 2 / 9 (22%) | 0 / 13 (0%) |  |
| No | 7 / 9 (78%) | 13 / 13 (100%) |  |
| **1^st^ Antipsychotic** |  |  | 0.6 |
| Low-potency antipsychotic | 0 / 9 (0%) | 1 / 13 (7.7%) |  |
| Atypical antipsychotic | 5 / 9 (56%) | 9 / 13 (69%) |  |
| No | 4 / 9 (44%) | 3 / 13 (23%) |  |
| **2^nd^ Antipsychotic** |  |  | >0.9 |
| Low-potency antipsychotic | 0 / 9 (0%) | 1 / 13 (7.7%) |  |
| Atypical antipsychotic | 1 / 9 (11%) | 2 / 13 (15%) |  |
| No | 8 / 9 (89%) | 10 / 13 (77%) |  |
| **Lithium** | 0 / 9 (0%) | 3 / 13 (23%) | 0.2 |
| **Mood stabilisators** |  |  |  |
| No | 9 / 9 (100%) | 13 / 13 (100%) |  |
| **Antidiabetics** |  |  | 0.7 |
| Insulin | 0 / 9 (0%) | 1 / 13 (7.7%) |  |
| Other | 1 / 9 (11%) | 0 / 13 (0%) |  |
| No | 8 / 9 (89%) | 12 / 13 (92%) |  |
| **Painkiller** |  |  | 0.4 |
| NSAID | 1 / 9 (11%) | 0 / 13 (0%) |  |
| No | 8 / 9 (89%) | 13 / 13 (100%) |  |
| **Antibiotic Intake** |  |  | 0.2 |
| Within last month | 2 / 9 (22%) | 0 / 13 (0%) |  |
| No | 7 / 9 (78%) | 13 / 13 (100%) |  |
| **PPI** | 2 / 9 (22%) | 4 / 13 (31%) | >0.9 |
| **Immunosuppressive** |  |  |  |
| No | 9 / 9 (100%) | 13 / 13 (100%) |  |
| **Laxative** | 1 / 9 (11%) | 1 / 13 (7.7%) | >0.9 |
| **Statins** | 2 / 9 (22%) | 0 / 13 (0%) | 0.2 |
| **Previous Surgery** |  |  | >0.9 |
| None | 9 / 9 (100%) | 11 / 13 (85%) |  |
| Bowel surgery | 0 / 9 (0%) | 1 / 13 (7.7%) |  |
| Other abdominal surgery | 0 / 9 (0%) | 1 / 13 (7.7%) |  |
| **Diagnosis** |  |  | 0.3 |
| F33.2 | 5 / 9 (56%) | 10 / 13 (77%) |  |
| F33.3 | 1 / 9 (11%) | 3 / 13 (23%) |  |
| F33.1 | 1 / 9 (11%) | 0 / 13 (0%) |  |
| F33.1 | 1 / 9 (11%) | 0 / 13 (0%) |  |
| F33.4 | 1 / 9 (11%) | 0 / 13 (0%) |  |
| **Ethnicity** |  |  | >0.9 |
| Caucasian | 9 / 9 (100%) | 12 / 13 (92%) |  |
| Other | 0 / 9 (0%) | 1 / 13 (7.7%) |  |
| **Diet** |  |  | 0.7 |
| Omnivorous | 7 / 9 (78%) | 12 / 13 (92%) |  |
| Vegetarian | 1 / 9 (11%) | 1 / 13 (7.7%) |  |
| Vegan | 1 / 9 (11%) | 0 / 13 (0%) |  |
| **Daily alcohol consumption** |  |  | >0.9 |
| Never | 8 / 9 (89%) | 11 / 13 (85%) |  |
| Formerly | 0 / 9 (0%) | 1 / 13 (7.7%) |  |
| Currently | 1 / 9 (11%) | 1 / 13 (7.7%) |  |
| **Smoking** |  |  | **0.080** |
| No | 7 / 9 (78%) | 4 / 13 (31%) |  |
| Yes | 2 / 9 (22%) | 9 / 13 (69%) |  |
| **MADRS pre-ECT (week 0)** | 14 (6) | 28 (7) | **<0.001** |
| **MADRS post-ECT (week 4)** | 13.8 (7.3) | 15.2 (6.2) | 0.6 |
| **Difference MADRS** | 1 (3) | 12 (8) | **<0.001** |
| **BDI-II pre-ECT (week 0)** | 26 (12) | 40 (9) | **0.021** |
| **BDI-II post-ECT (week 4)** | 23 (12) | 19 (15) | 0.3 |
| **Difference BDI-II** | 3 (6) | 20 (16) | **0.008** |
| *^1^*Mean (SD); n / N (%) | | | |
| *^2^*Mann-Whitney U test; Fisher's exact test | | | |

**Supplementary table 1**: Clinical characteristics electroconvulsive therapy (ECT) patients and controls

BDI-II – Beck Depression Inventory, BMI – Body mass index, MADRS - [Montgomery–Åsberg Depression Rating Scale,](https://de.wikipedia.org/wiki/Montgomery%E2%80%93%C3%85sberg_Depression_Rating_Scale) SSRI – Selective serotonin reuptake inhibitors, SSNRI – Selective Serotonin-noradrenaline reuptake inhibitors, NSAID – Non-steroidal anti-inflammatory drugs
